# Developing a mental health care plan in a low resource setting: the theory of change approach

**DOI:** 10.1186/s12913-015-1097-4

**Published:** 2015-09-28

**Authors:** Maji Hailemariam, Abebaw Fekadu, Medhin Selamu, Atalay Alem, Girmay Medhin, Tedla Wolde Giorgis, Mary DeSilva, Erica Breuer

**Affiliations:** Department of Psychiatry, School of Medicine, College of Health Sciences, Addis Ababa University, PO Box 9086, Addis Ababa, Ethiopia; King’s College London, Institute of Psychiatry, Department of Psychological Medicine, Centre for Affective Disorders and Affective Disorders Research Group, London, UK; Aklilu Lemma Institute of Pathobiology, Addis Ababa University, Addis Ababa, Ethiopia; Federal Ministry of Health, Addis Ababa, Ethiopia; Centre for Global Mental Health, London School of Hygiene and Tropical Medicine, London, UK; Alan Flisher Centre for Public Mental Health, Department of Psychiatry, University of Cape Town, Cape Town, South Africa

**Keywords:** Integrated care, Complex intervention, Ethiopia, Theory of change, Mental health care plan

## Abstract

**Background:**

Scaling up mental healthcare through integration into primary care remains the main strategy to address the extensive unmet mental health need in low-income countries. For integrated care to achieve its goal, a clear understanding of the organisational processes that can promote and hinder the integration and delivery of mental health care is essential. Theory of Change (ToC), a method employed in the planning, implementation and evaluation of complex community initiatives, is an innovative approach that has the potential to assist in the development of a comprehensive mental health care plan (MHCP), which can inform the delivery of integrated care. We used the ToC approach to develop a MHCP in a rural district in Ethiopia. The work was part of a cross-country study, the Programme for Improving Mental Health Care (PRIME) which focuses on developing evidence on the integration of mental health in to primary care.

**Methods:**

An iterative ToC development process was undertaken involving multiple workshops with stakeholders from diverse backgrounds that included representatives from the community, faith and traditional healers, community associations, non-governmental organisations, Zonal, Regional and Federal level government offices, higher education institutions, social work and mental health specialists (psychiatrists and psychiatric nurses). The objective of this study is to report the process of implementing the ToC approach in developing mental health care plan.

**Results:**

A total of 46 persons participated in four ToC workshops. Four critical path dimensions were identified: community, health facility, administrative and higher level care organisation. The ToC participants were actively engaged in the process and the ToC encouraged strong commitment among participants. Key opportunities and barriers to implementation and how to overcome these were suggested. During the workshops, a map incorporating the key agreed outcomes and outcome indicators was developed and finalized later.

**Conclusions:**

The ToC approach was found to be an important component in the development of the MHCP and to encourage broad political support for the integration of mental health services into primary care. The method may have broader applicability in planning complex health interventions in low resource settings.

## Background

The burden of mental disorders on individuals and society in low income settings is substantial [1, 2]. At least one in 10 adults is affected by a mental disorder. In some low income countries, nine in 10 individuals with a mental disorder do not access even basic treatment [3, 4]. Although the need for improving services is clear, numerous barriers to service provision remain unaddressed [5, 6]. Mental health care is given minimal policy attention and the human and organisational resources available are inadequate and inefficiently utilized [7, 8]. As a consequence, the mental health treatment gap remains large [9].

Currently, there are few studies carried out to determine the prevalence of mental disorders in Sodo district, Southern Ethiopia. A study conducted in the neighbouring district indicated that the lifetime prevalence of schizophrenia is 0.5 % [10]. In another study conducted in Sodo, the one month prevalence of common mental disorders for the mild, moderate and severe was 13.8, 9.0 and 5.1 % respectively [11]. The study also indicated that hazardous alcohol use is common with 22.4 % prevalence [11]. There is a large treatment gap in Ethiopia for different mental disorders. For instance, over 90 % of cases with psychosis remain untreated [9].

The integration of mental health care into primary care has been the commonest approach advocated to narrow the treatment gap in low income countries since the 1970’s [12–14]. To assist this integration the WHO has provided evidence-based templates of intervention called the Mental Health Gap Action Programme (mhGAP) intervention guide [15]. The mhGAP provides a minimum set of evidence-based interventions that could be provided at the primary care level.

Nevertheless, the mhGAP does not provide guidance on how mental health care can be adapted to and integrated into primary care within an Ethiopian setting [16]. This general lack of evidence on the feasible, acceptable and effective ways of integrating and scaling up of mental health care is a reflection of the complex nature of integration. The integration of mental healthcare into primary care requires careful planning, stakeholder buy in, and active community participation. There is a need for an innovative approach to plan, implement and evaluate the integration of mental health care into primary care.

The PRogramme for Improving Mental health carE (PRIME) is a cross-country mental health services research initiative that includes Ethiopia, India, Nepal, South Africa and Uganda. The primary aim of the project is to provide a robust evidence base on how mental health care can be integrated into primary care in low resource settings. In Ethiopia, PRIME works to develop services for selected priority disorders comprising psychosis, depression, epilepsy and alcohol use disorders. As part of the PRIME project undertaking, the Ethiopian team conducted Theory of Change (ToC) workshops. The workshops were designed to understand the complex interaction of contextual factors in the delivery of mental health care. Employing workshops was also important to map out the chain of inter-dependent pre-conditions necessary for the delivery of mental health care [17]. The ToC approach has been used rarely in mental healthcare planning. Therefore, before describing the ToC study methods, we would start by defining the ToC approach.

ToC is “a theory of how and why an initiative works” [18] which makes explicit the short-and medium- pre-conditions and long-term outcomes required to achieve the impact of a complex intervention [19, 20]. ToC is displayed as a causal pathways map with a number of key elements.

After defining the status of how things operate before the start of the programme, pre-conditions (also called short term outcomes) on the pathway to impact are mapped out. The mapping took a causal sequence leading to the long-term outcomes of the programme.

**Fig. 1:**
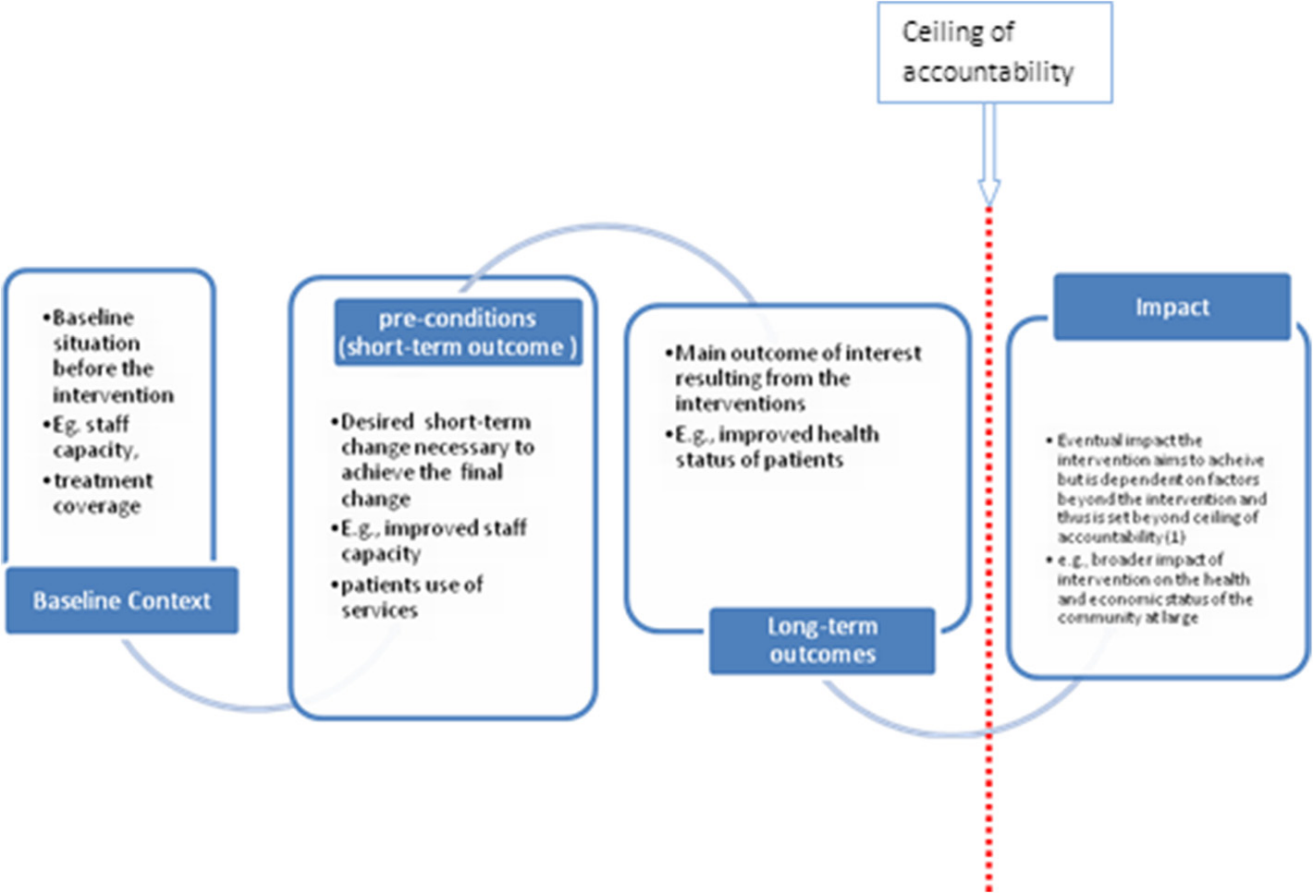
The ToC approach

For instance, availability of the healthcare workers was set as a pre-condition 1 or short-term outcome 1. This pre-condition is followed by another pre-condition which is that the health workers have skills to diagnose and detect mental disorders (pre-condition or short-term outcome 2). The long-term outcome of the programme stated that the care provided by these skilled staff leads to improvement in the clinical status of treated patients. Interventions are used to move from the current status to the next pre-condition (e.g. training of health workers is needed to move from health workers availability to health workers’ skills to deliver care). Assumptions articulate the main barriers which need to be overcome to move through the pathway (e.g. general health workers are willing to be trained in mental health skills). Rationale states the evidence base for the pathway (e.g. evidence from clinical trials has shown that non-specialist health workers can deliver effective mental health care). The pathway is evaluated through the development of indicators for each pre-condition on the pathway (e.g. at least 2 general health workers in each primary health centre have the skills to appropriately diagnose and treat mental illness) [21, 22]. Fig. 1 describes how planning of a desired long-term outcome works taking into account the existing context. The necessary chains of shorter-term pre-conditions required to achieve the eventual desired goal or long-term impact are also described.

Demarcation between long-term outcomes and impact of the programme is essential. A ceiling of accountability marks the dividing line between specific long-term outcomes and the broader impact of the programme. PRIME will be held accountable for the specific long-term outcomes only. Nevertheless, the intervention by the programme might lead to the realisation of the broader impact of the programme. Hence, the impact of the programme could be translated as a broader outcome for which the programme will not be held accountable for.

The ToC map is developed iteratively through collaboration with key stakeholders in a series of ToC workshops. These workshops are conducted with a range of stakeholders early on in the planning process to identify key stages in the pathway. Workshops also elicit feasible and acceptable interventions that are needed to move along the pathway, and to clarify key assumptions about the context. A key goal of the workshops is to elicit stakeholder buy in by intimately involving them in the development of the intervention. Unlike focus group discussions or in-depth Interviews, which aim to elicit information based around topic guides, ToC workshops identify the pathway of change together with stakeholders thus obtaining stakeholder buy in and input into planning the intervention from beginning.

The ToC approach can be used for planning, implementation and evaluation of complex interventions [22–24] as it allows rigorous thinking and is an empowering approach that facilitates stakeholder participation starting from the planning stage [19, 17]. In the present study, we report about testing its use as a viable strategy for developing a pragmatic, community-based mental health care plan for the Sodo district in Ethiopia. This paper describes the process and effectiveness of the ToC process in Ethiopia to formulate a comprehensive district mental health care plan.

## Methods

### Setting

The study was conducted in the Sodo district in Southern Ethiopia, located about 100 km south of the capital city, Addis Ababa. It is the largest and, with a population of 161,000 [25], the second most populous part of the Gurage zone. The Sodo district is comprised of 58 Kebeles (the smallest administrative unit) which are both geographically and climatically diverse. The majority of the population in the district are from the Gurage ethnic group and followers of Orthodox Christianity. The official language of the district is Amharic.

The district has eight health centres, four of which are located within the three towns of the district. Each health centre serves a population of about 25,000–40,000 people [26, 27]. Most people in the district are within an hours’ walking distance of a health centre. The lowest statutory healthcare facility is the health post, located in each kebele or sub-district. The 58 health posts in the district are staffed by a pair of community health workers called health extension workers (HEWs): high school graduates with one year training. The nearest general hospital which is also the nearest psychiatric facility, is located in a neighbouring district in Butajira town, about 35 km from Bui, the capital of Sodo district. There are no mental health services in Sodo district, and the primary care staff members receive very limited training in providing mental health care.

### Theory of change workshops

Prior to the ToC workshops in Ethiopia, the PRIME team:

1. Made several visits to the district and held meetings with the district administration.
2. Reviewed documents about the local health care system, including health centre registry, prescriptions, drug list, HMIS and monthly report summaries.
3. Conducted 10 exploratory interviews with leaders of the district.
4. Conducted a situational analysis of the health service context of the district which included data on relevant context, mental health policies and plans, mental health treatment coverage, district level health services, community and monitoring and evaluation.

The exploratory interviews conducted did not follow the typical qualitative interview approach. They were conducted just to understand the underlying situation in the district. The information provided was used to have a picture of the health care situation of the district only. No part of the interviews was used to supplement the ToC approach.

In consultation with the district administration, the PRIME team identified participants for the ToC workshops representing different segments of the society. A facilitator trained in the use of ToC moderated the workshops. The major role of the facilitator was to introduce and describe the TOC approach, objectives of PRIME, and findings of the initial situational analysis. Throughout the workshop, the facilitator moderated discussions around pre-conditions, indicators, assumptions and outcomes of the intervention.

Current state of the district and the final agreed outcome of the programme were written at the left and right ends of a whiteboard. Participants were asked to suggest causal pathways projecting from the current state and leading to the final outcome of the programme. Four levels of intervention involving the community, health care facility, health care administration and higher policy organizations were indicated. The final outcome of the programme was defined as improved health, social and economic outcomes for people with priority mental disorders in the district. To achieve this, participants were requested to state the required pre-conditions, interventions, assumptions and indicators. Along the pathways, sets of interlinked pre-conditions were connected to the next pre-condition through interventions and assumptions. For each intervention, indicators were identified and agreed up on. All of the participants contributed their share in refining the pathways to the final outcome.

**Fig. 2:**
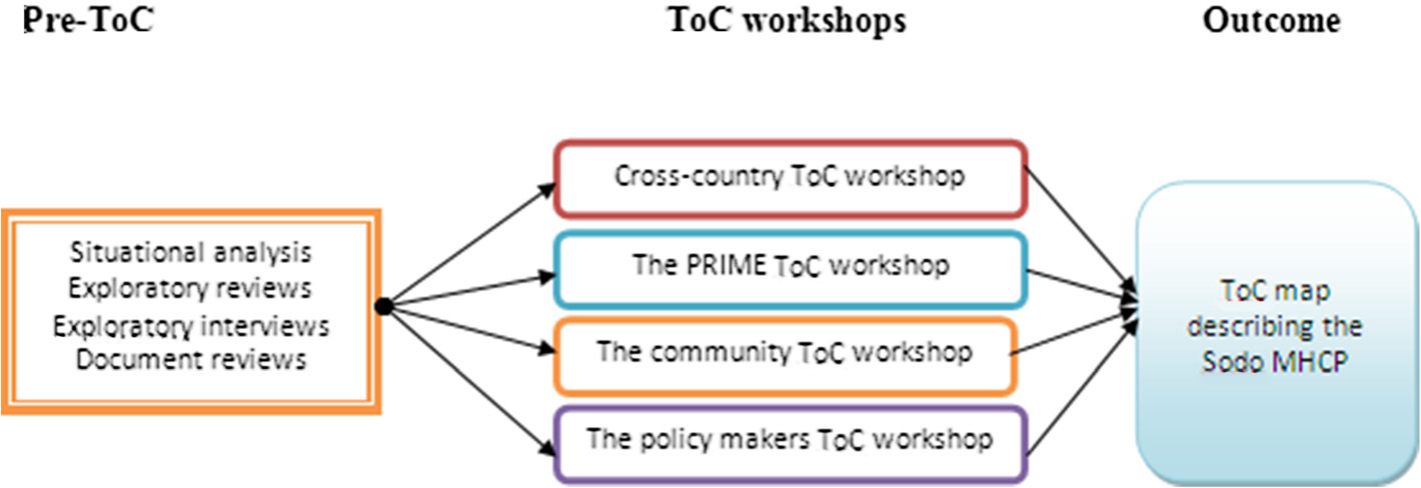
The ToC Process in developing the Sodo district mental health care plan

Four ToC workshops were held with various stakeholders from within and outside the district. Workshops were held: 1) at the cross-country level; 2) within the PRIME Ethiopia team; 3) with policy-makers; and 4) with Community representatives. The cross-country ToC workshop was held across all PRIME countries to start to develop a generic ToC map [17]. This paper focuses specifically on the Ethiopian ToC process attempting to describe contextual issues and resources. The Ethiopian ToC workshops aimed at understanding context specific factors with respect to each of these groups included in the workshop. To ensure relative homogeneity of participants, each of the Ethiopian workshops was held separately. Except for the cross-country workshop, which was carried out in September 2011, all the three workshops were held over 2 weeks period from 25 January-11 February 2012. This process is illustrated in Fig. 2.

Results from the pre-ToC work and the ToC workshops were combined to develop a ToC map for the proposed MHCP in Sodo district.

### Theory of change workshop participants

Participants of the ToC workshops were selected purposively on the basis of their involvement in mental health policy and service planning; expertise in the area of mental health care and social programmes; involvement with the community as community members or community leaders. Three groups of people were involved in the workshop: 1) federal and local policy makers; 2) mental health experts and researchers; and 3) community representatives. To ensure a balanced representation of stakeholders in the planning process, different sectors such as justice, health facility, women and youth representatives, and faith and non-statutory leaders were part of the workshops. Attempts were made to ensure fair representation of women in the workshops and their participation during the discussions was highly encouraged. Details of the cross-country ToC is described elsewhere [17].

Three in-country ToC workshops with 46 participants were run over 2 weeks period. The PRIME ToC workshop included 10 people who were involved in research and administrative activities of the project. The community ToC involved 20 people from the community and the district administration, who were also involved in the Community Advisory Board (CAB) for the PRIME project in Ethiopia. The CAB was composed of members of the district administration, police, health, representatives of faith and traditional healers, women’s and children’s affairs and youth associations. Finally, the policy makers’ ToC involved 16 people representing the Federal MoH, mental health professionals, social workers, district and zonal health bureau representatives. Five PRIME team members attended all three workshops. Minutes from each of the workshops were reviewed and used by the PRIME team to finalise the ToC map after all the workshops were held. Pre-conditions, long-term outcomes, interventions, assumptions and indicators were displayed on the ToC map. Inputs from the situational analysis and exploratory interviews were also used to enrich the ToC map further.

### Data collection

Minutes were captured by two PRIME staff members (MH and MS) with a masters degree in social work. Minute takers were trained on the basic principles of the ToC approach prior to the workshops. All workshops were audio taped and final minutes were checked against the audio file for completeness. Registration forms with basic information on education, work experience in relation to mental health were also administered to all participants.

### Ethical consideration

Ethical clearance of the study was obtained from the Institutional Review Board, College of Health Sciences of Addis Ababa University. All participants provided informed consent. By its nature, information obtained was in a group setting and members knew each other. No personal information was obtained from the participants. Information collected was also anonymous.

## Results

### Socio-demographic characteristics

A total of 46 participants representing diverse stakeholders were involved in three ToC workshops. All of the participants were working age adults. The academic backgrounds of participants ranged from non-literate (in the community group) to higher level mental health professionals including clinical psychologists, public health professionals, social workers and psychiatrists (in the policy makers’ group). Except the participants of the policy makers ToC workshop and the PRIME team members, all of the participants were based in the district.

**Fig. 3:**
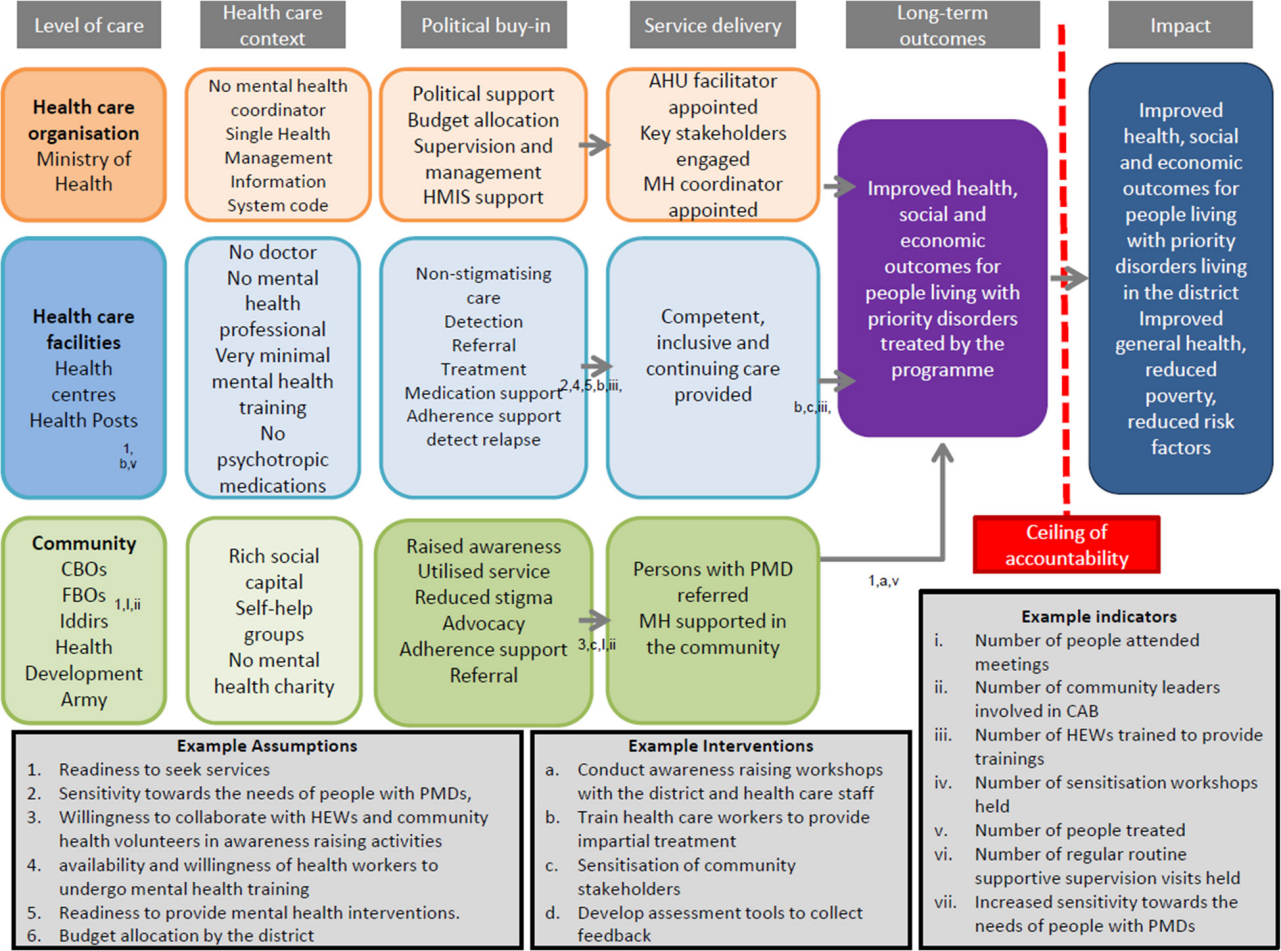
The ToC map

The following section describes details of the ToC map including the key pre-conditions, the pathways at the different levels of care, the interventions and the indicators. Figure 3 presents abridged version of the ToC map for the MHCP in Sodo.

### Key pre-conditions in the ToC map

Four distinct dimensions comprising the community, health care facility, district and higher (federal) level health care administration were identified. Various distinct pre-conditions were identified for these levels. These different pre-conditions were identified by stakeholders as the essential milestones in the path towards integrated mental health care at the primary care level. Achieving these pre-conditions would lead to the targeted long-term outcome: improvement in health, social and economic status of people using mental health services. The long-term outcome of the programme was established during the cross-country workshop. Later, it was presented as a discussion point during the in-country ToC workshops and approved by all stakeholders. The broader impact (for which PRIME would not be held accountable) included a reduction in crime, domestic violence and unnatural deaths in the community. The pre-conditions at the different levels are described below. The required pre-conditions were mapped following the three intervention lines. These included the community, health facilities, district healthcare administrations and higher health care administration (Zonal health bureau, regional health bureau and the Federal Ministry of Health.

### Community level pathways

The baseline situation in Sodo was discussed in all the ToC workshops. The existing coverage of mental health services in the community was reported to be very low. As identified during the workshops, most people with severe mental disorders in the district were reported to have been chained up or shackled at home. The underlying assumption in the district is that mental disorders were caused by possession by evil spirits. Therefore, the common practice is that they remain untreated or often receive help only from traditional healers. The few people known to seek care travelled to urban centres notwithstanding high costs of transportation and accommodation.

Findings from the situational analysis and exploratory interviews suggested that there was no mental healthcare service in the district. Yet, as a long-term outcome, the programme aspires to see improvement in the health, social and economic status of people with priority disorders. Hence, the ToC workshops with the community were aimed at filling out the empty space between the current state and the long-term outcome with possible short-term pre-conditions, indicators and assumptions. At some points during the workshop, differences in opinion and divergence in recommendations were observed. In those cases, both ideas were forwarded to the group for discussion until consensus was reached. Disagreements also emerged where there was lack of information. Such topics were recorded for further exploration after the ToC process.

**Fig. 4:**
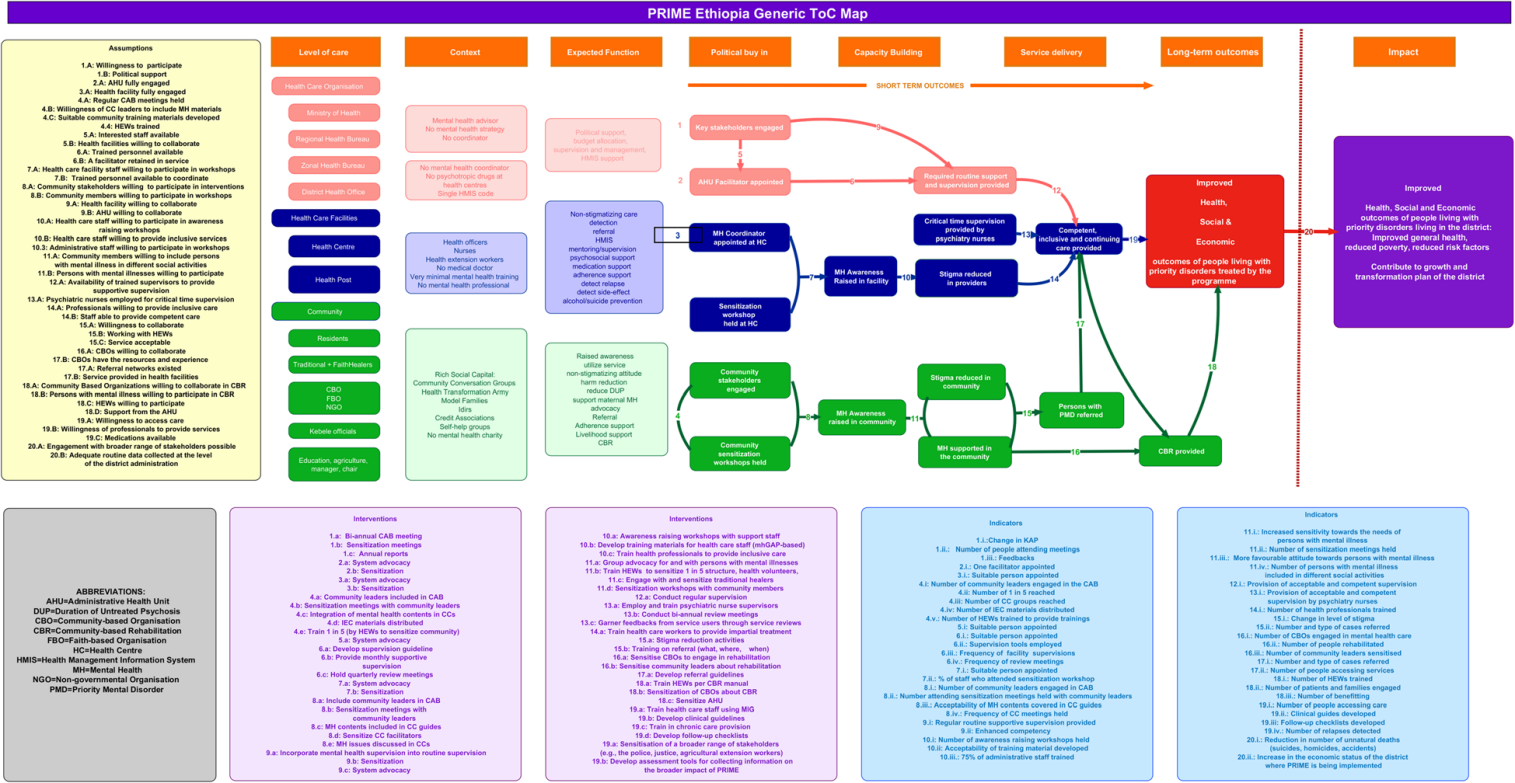
The final ToC map

Participants were requested to list assumptions, interventions and indicators. These three were numbered written in a separate flipchart. Numbers and letters representing the assumptions, interventions and indicators were used to link one pre-condition to the next one. Possible interventions and indicators to measure success of the project were jointly set with the community workshop participants. Details of the process are illustrated in Fig. 4.

To achieve the long-term outcomes, the current lack of awareness about mental health at the community level was mentioned as being problematic. Therefore, raised mental health awareness and increased help seeking behaviour among people with priority disorders were identified as possible short-term outcomes. The provision of adherence support by families, HEWs and community volunteers was also suggested to strengthen service delivery. The current situation also indicated that HEWs did not have the skills to detect and refer cases they suspected to be mental disorder. Therefore, improving their skills to detect and refer people with potential mental disorders was recommended as crucial. As stated during the workshops, HEWs may be helpful in improving adherence, detecting relapse and providing family support.

Apart from reliance on HEWs for detection and referral, sensitizing and engaging with broad range of community stakeholders was also mentioned essential. To this end, working with community based organisations (CBOs), community leaders, teachers, health development army (a group of community members who work as health volunteers), other extension workers (e.g., agricultural extension workers), traditional healers, non-governmental organisations (NGOs) and faith based organisations (FBOs) was mentioned instrumental in achieving community level pre-conditions.

Strengthening the existing community initiatives to increase mental health awareness and support persons with mental disorders was indicated as an important intervention to achieve these pre-conditions. Engaging with persons with mental disorders and their families in different community activities and providing community based rehabilitation (for people with more severe disorders) were reported to be vital. Involvement of CBOs, FBOs and NGOs in different social and economic activities was reported to have a direct bearing on stigma reduction and the promotion of social inclusion of people with mental disorders.

The key long-term outcomes (clinical, social and economic) described in the ToC map relate to the community pathways. Improved outcome for individual patients and their families in these areas would represent both individual, family and community outcomes. Although distinguishing between social and economic outcomes was considered difficult, change in one is likely to lead to change in the other. Reduced stigma in the community or in work places were identified as both social and economic outcomes. This is potentially because of the direct impact of reduced stigma on the social and economic conditions of people with mental disorders.

### Facility level pathways

The situational analysis identified eight health centres and 58 health posts that were operating in the district during the time of the assessment. All of these health facilities were established by the government. Health centres were staffed by nurses, laboratory technologists, pharmacists, pharmacy technicians and supporting staffs. The health posts were staffed by HEWs. None of the facilities have trained mental health personnel. No mental health care provision was reported in any of these facilities. Cases with mental health problems in the community were referred to the capital city, Addis Ababa.

The key pre-conditions for clinical staff at the facility level were the attainment of adequate levels of competence in case detection, treatment and monitoring of care and the provision of inclusive care for persons with mental disorders. At the facility level, it was recommended that the health care staff should diagnose, treat, manage drug side-effects, and support adherence. Primary health care staff should also refer persons with mental disorders for community-based support, including income generating activities, provide psychosocial support, assess social needs and provide recovery education.

### District health care administration level pathways

A proper reporting framework with clear mental health indicators did not exist at the district level. Psychotropic medications were not included in the current drug list of the district. The district officials also mentioned that lack of mental health awareness is common among the different officials in the district.

Allocation of budget, facilitation of resources and personnel for training, raised awareness and demonstration of political commitment were suggested as expected pre-conditions from the district health care administration. Developing non-stigmatising attitudes and engaging in advocacy initiatives which promote the inclusion of persons with mental disorders in different social activities within and beyond the district were also highlighted. Supporting the implementation of economic policies for persons with mental disorders and their families to improve their economic outcomes was emphasised as being essential for the MHCPs. In addition, it was emphasised that the district should encourage different development organisations to involve persons with mental illness in their activities. The importance of political commitment to support economic wellbeing of persons with mental illness and their families was also mentioned as significant. Although these activities are important for the successful rehabilitation programmes, the MHCP focused only on those activities that were feasible and affordable within the constraints of funding by the MoH.

### Higher healthcare administration level pathways

At the national level, the national mental health strategy has been developed, endorsed and is pending its implementation [28]. Most of the suggested outcomes at the health care organisation level are related to raising mental health awareness at the health care organisation level and budget allocation. The need for strong political commitment was also emphasised. The development of a national mental health strategy is taken as indicative of the political commitment of the Federal MoH of Ethiopia.

Raised awareness at the MoH was mentioned as having a direct impact on budget allocation and on the inclusion of mental health in the national Health Management Information System (HMIS). Obtaining political buy in was mentioned as vital for the development of a successful MHCP.

### Assumptions and indicators

In addition to the causal pathway of pre-conditions leading to long-term outcomes, assumptions and indicators were also highlighted during the workshops. At the community level, the underlying assumptions included readiness to seek services among people with mental health problems, sensitivity of the general public towards the needs of people with mental disorders, and willingness to collaborate with HEWs and community health volunteers in awareness raising activities. Facility level assumptions included the availability and willingness of health workers to undergo mental health training and their readiness to provide mental health interventions. The political commitment of the district and health care administration, raised mental health awareness, and adequate budget allocation were cited as assumptions at the district and higher health care administration levels, which are necessary to make the MHCPs effective.

For all the pre-conditions outlined in the ToC, indicators were developed to enable a comprehensive evaluation of the process of implementation and the impact of the MHCP. Indicators were identified for the long-term outcomes of improved health, social and economic outcomes for people treated by the MHCPs. Health outcome indicators listed were improvement in symptom levels and in functional status. Indicators of improved social and economic outcomes included active engagement of people with mental disorders and their families in the different social affairs of their community, and improvement in the economic conditions of people with mental disorders and their families (Details are included in the ToC map, Fig. 3).

Additional facility and district level indicators of political commitment included the appointment of a separate mental health professional at the district level to coordinate mental health services in the district. Capacity building and ratification of policies for the inclusion of people with mental disorders at different development activities were also mentioned as policy level indicators.

## Discussion

The ToC approach has the potential to be used as an important framework in developing mental health care plan in low-resource settings. Developing a mental health care plan in these settings requires rigorous development of interventions and a broad-based support from the community and policy makers. Meticulous thinking, explaining and articulating the theory behind an intervention is a prerequisite for effective mental health care planning, as well as for planning other complex interventions.

The approach combines the dynamic complexity of interventions within the diversity of settings by taking in to account the objective realities at the grass-roots level [29, 30]. For the work in the Sodo district, the ToC approach was found to be a very useful approach to elicit wide participation in planning mental health services. The ToC process played a key role in achieving political commitment and understanding the awareness levels of various stakeholders involved in the workshops. In doing so, it also made the mental health care plan of the Sodo district more feasible and improved the chance of it being effective and sustainable.

The active participation of multiple stakeholders in planning complex community interventions is important to increase the effectiveness of interventions by creating a sense of ownership [31, 32]. Strong community participation is also a key aspect in developing suitable interventions that take into account the peculiarities of the settings. The ToC process considers the geographic, social and political context in which the mental health care intervention will take place.

A cross-country evaluation to the PRIME ToC workshops indicated that heterogeneity of participants might hinder balanced participation [17]. We would like to confirm that heterogeneity is a challenge for any study conducted in a community setting. Particularly, in studies like the present study which included wide-range of stakeholders, variation in participation could be noticed. For instance, it was observed that traditional healers participated less often compared to the other participants. This could be attributed to their lack of trust in the process or their discomfort with the heterogeneity of the group. Nonetheless, heterogeneity could not be completely eliminated as a factor in how participants contribute to the planning process. To ensure relative heterogeneity, dividing the participants in to three separate groups and conducting a separate ToC workshop was essential.

The ToC approach was found to be an effective mechanism to engage with and obtain buy in of multiple stakeholders. It provides interventions with a strong community-base and also to ensure the sustainability of outcomes [32, 33]. In addition, through our ToC workshops, we have learned that engaging the community in service planning is critical in terms of understanding the context in which the intervention will take place [34].

The responsiveness, quality and strength of the mental health care plan is determined by the even representation and genuine participation of all stakeholders throughout the planning process [20]. To this end, we have understood that the involvement of diverse stakeholders has contributed to informing and involving potential actors of the intervention. The fact that we have involved stakeholders from various government and community organisations has contributed to the development of a comprehensive mental health care plan. In addition to these, their involvement also enabled us to identify the fact that the community is rich in social capital and has wide projecting social networks.

The subsequent meetings held with different officials within the district prior to the workshops also enabled us to assess and more accurately identify the specific gaps in mental health treatment in the district. As part of the ToC workshops, we have learned that there is dearth of trained human resources for mental health care.

The mhGAP works best in health care settings where doctors are available. The approach fails to give a clear guideline as to how to adopt the interventions for settings without doctors. If MhGAP interventions are to work in contexts where there are no doctors, there is a need for special adaptation of the MhGAP to work with other primary care workers like the health officers and nurses. Thus, understanding the context through the ToC approach is a viable strategy to ensure effectiveness of mental health care planning. To this end, we strongly recommend the ToC as a tool for understanding the context of any service delivery.

Our ToC workshops also suggested that the involvement of key policy actors in the planning process has some added benefits. The ToC workshop with policy makers allowed us to understand the broader national context such as high level of commitment of the MoH to integrating mental health care into primary care. In 2012, The Ethiopian MoH endorsed a national mental health strategy [28]. In addition, we established that the findings of exploratory interviews regarding the health service context of the district were in line with the health care system of the country which is very much decentralised [27].

At more grass-roots level, understanding what the people in Sodo think about mental disorders, choices and decisions they make, economic constraints and broader socio-cultural factors are important for service planning. Although these factors play a key role in determining help seeking, our workshops did not probe in to these directly. Qualitative exploration would have been ideal to understand some context specific factors. In this study, we have not attempted to supplement the ToC study with qualitative explorations. Although this is an important limitation, we presented the ToC as an independent approach to define and understand interventions of a complex nature.

We recommend that the TOC is an important approach to use in low-resource rural settings such as the Sodo district. Other studies have explored the local terminologies and explanatory models of mental disorders [35–38]. Generally, only severe mental disorders are considered as illnesses while milder forms of mental disorder, such as some types of depression are unrecognized. These disorders are typically attributed to supernatural causes and treatment is sought in the first instance from traditional providers who are believed to have the skills to deal with predicaments caused by supernatural causes.

Although the ToC workshops were extremely informative in terms of understanding how the healthcare system works, the approach hardly gives clear guide on every detail [22]. For instance, some of the questions raised about the HMIS remain unanswered during the workshop. The ToC workshop is designed to be a consensus building exercise than a comprehensive data collection technique. As a result, follow-up interviews were needed to enhance understanding of some structural factors including HMIS. Instead, the group built the ToC map through a process of discussion, reflection and ultimate consensus through resolution of differences, so detailed data on any disagreements and resolutions were not captured.

Another limitation of the method emanates from the fact that all the assumptions and indicators are developed at the very beginning of the process. Consequently, the approach failed to capture pragmatic challenges that might emerge during implementation. Therefore, to refine the pathway of change and enhance elasticity of the ToC map, follow up ToC is required. ToC maps provide an essential path but could not be considered as a comprehensive guide [23]. Nevertheless, other studies confirmed that the ToC process enables intervention designers to maximize opportunities for research and establish networks with the community [39].

### Limitations

Since the ToC approach is a new concept for the workshop participants, it was necessary to provide a framework highlighting the pathways developed by the PRIME team. Thus, the input of participants might have been influenced by what was already being provided. Nevertheless, to the satisfaction of the investigators, the input of the participants was often original and detailed. Although we have included community representatives as part of the ToC process, we were unable to include service users from the community. Our primary intent was to engage service users from the district who were accessing mental health care in a primary care facility during the time of the study. However, since mental health service was not available, we were unable to find such persons during the time of the study. Those service users whom we have invited from the neighbouring district who were accessing care in a hospital did not show up due to an undisclosed reason. A carer who was invited to the policy makers workshop also did not attend. Another limitation of the ToC process was that we were not able to involve representatives from the regional health bureau. As a result, some of the issues that required their decision remained open for further exploration after the ToC.

## Conclusions

Employing the ToC approach to mental health care planning is an effective approach to developing a pragmatic and community based mental health care plan. The involvement of multiple stakeholders at different levels served as a pathway to elicit stakeholder buy in, helped planners to define assumptions from the start of the process, and reduced uncertainties about the implementation. The approach may serve as an important framework in identifying the necessary and sufficient conditions to make the intended long-term outcomes of the programme a reality.

## Abbreviations

AHU: Administrative health unit
CAB: Community advisory board
CBO: Community based organisation
CBR: Community based rehabilitation
DFID: Department for international development
FBO: Faith based organisation
HEW: Health extension worker
HMIS: Health management information system
LAMIC: Low and middle income countries
MoH: Ministry of health
MHCP: Mental health care plan
mhGAP: Mental health gap action programme
NGO: Non Governmental Organisation
PRIME: Programme for improving mental health carE
SMD: Severe mental disorders
SNNPRS: Southern Nations, Nationalities and People’s Regional State
ToC: Theory of change
WHO: World Health Organisation
